# Trope analysis and folk intuitions

**DOI:** 10.1007/s11229-020-03013-3

**Published:** 2021-01-11

**Authors:** Stephanie Rennick

**Affiliations:** Department of Philosophy, University of Glasgow, 69 Oakfield Avenue, Glasgow, G12 8QQ UK

**Keywords:** Folk intuitions, Imagination, Tropes, Fiction, Methodology

## Abstract

This paper outlines a new method for identifying folk intuitions to complement armchair intuiting and experimental philosophy (X-Phi), and thereby enrich the philosopher’s toolkit. This new approach—trope analysis—depends not on what people report their intuitions to be but rather on what they have made and engaged with; I propose that tropes in fiction (‘you can’t change the past’, ‘a foreknown future isn’t free’ and so forth) reveal which theories, concepts and ideas we find intuitive, repeatedly and *en masse.* Imagination plays a dual role in both existing methods and this new approach: it enables us to create the scenarios that elicit our intuitions, and also to mentally represent them. The method I propose allows us to leverage the imagination of the many rather than the few on both counts—scenarios are both created and consumed by the folk themselves.

## Introduction

In this paper I propose a new method for identifying folk intuitions by analysing artefacts of the popular imagination. This new approach (‘trope analysis’) is a complement to traditional methods of intuition gathering—philosophising from the armchair and experimental philosophy (X-Phi)—adding a new tool to the philosopher’s toolkit. I remain neutral as to the precise nature of intuitions, suffice to say that whatever one takes the output of the existing approaches to be, the same sort of thing can be gleaned via trope analysis. In Sect. 2, I sketch how folk intuitions are used in philosophy and then in Sect. 3 outline two existing methods for their identification. In Sect. 4, I introduce trope analysis with discussion of its mechanics, advantages and novelty in Sects. 4.1, 4.2 and 4.3, respectively. Finally in Sect. 5, I tackle potential objections to the use and usefulness of the approach.

## The importance of folk intuitions

Folk intuitions are frequently mentioned and made use of across philosophy, from discussions of the nature of pain, to the rightness of an action, to the distinction between knowledge and belief. They are employed as a starting point for conceptual analysis, to verify that all parties are talking about the same (or intended) thing, and claimed as evidence in favour of a view (that a particular theory corresponds with ‘common sense’ or the view ‘of the person on the Clapham omnibus’ is often thought to weigh in its favour).

The debate over what constitutes free will is an illustrative example: it is particularly prone to arguments about folk or pre-theoretical intuitions, with philosophers in various camps claiming that the folk are on their side. This claim is a powerful one: there is an oft-cited passage by Mele (2001: p. 27) in which he writes that any adequate analysis of free will and related notions should be “anchored by common-sense judgements”, because if our analysis of what free will is and when we have it is at odds with the way ordinary people use the words and conceive of the notion, our analysis “runs the risk of having nothing more than a philosophical fiction as its subject matter.” Similarly, Jackson (1998: p. 31) writes that the free will debate should centre on our ordinary conception, which we can identify by appealing “to what seems to us most obvious and central about free action… as revealed by our intuitions about possible cases.”

Folk intuitions aren’t the end of the story; they aren’t conclusive evidence as to the rightness or wrongness of a theory. But they are a common starting point, they help us keep our discourse relevant to the initial concerns, and

if a philosophical theory *does* turn out to be privileged by the endorsement of the folk, that would seem to position the burden of proof on the shoulders of those who argue *contrary* to folk intuitions” (Nahmias et al. 2005: p. 564).

These attitudes are not restricted to discussions of free will:

Minimally, any philosopher who offers an account of intentional action that is not anchored by folk judgments would need to offer an error theory that explains how and why the folk are misapplying the concept (Nadelhoffer 2004: p. 196).

Some are more willing than others to embrace philosophical theories that are at odds with our pretheoretical conception of the world. But virtually everyone agrees that, even after having presented the arguments for their positions, proponents of revisionary philosophical theories—that is, those that deviate from the pretheoretical conceptions—are required to provide some sort of account of the conflict between their theories and the pretheoretical beliefs of non-philosophers (“the folk”) (Korman 2009: p. 242).

And,

Philosophy is standardly viewed as relying on *intuition* as a source of evidence for or against philosophical claims or theories. A successful philosophical theory of (say) knowledge is expected to align with our intuitions about knowledge—and often rather precisely, too (Nado 2014: p. 631).^1^

Whether or not these are the appropriate ways to use folk intuitions, or whether they exhaust their potential, is not the focus of this paper.^2^ Instead, acknowledging the status quo, I propose a new way of accessing folk intuitions.

## Current methods

Generally speaking, there are two main ways that philosophers identify the intuitions of the folk: musing from ‘the armchair’, and experimental philosophy (X-Phi).^3^ Each has its strengths and weaknesses, and nothing I have to say here is tantamount to a rejection of either—my purpose is to add an additional tool to the intuition seeker’s toolkit. I will remain neutral as to the precise nature of intuitions—whether they are, for instance, beliefs (Goldman and Pust 1998; Devitt 2006; Lewis 1983); inclinations or dispositions to believe or judge (Earlenbaugh and Molyneux 2009); a “*sui generis* kind of seeming” (Goldman 2007: p. 7; cf. Nagel 2007; Chudnoff 2011) etc. Whatever one takes the folk intuitions accessed from the armchair or X-Phi to be, so too can they be captured by this new approach (see Sect. 4 for more on this).

The existing methods are not completely distinct from one another. We might be tempted to think of the armchair as in some sense a priori and X-phi as a posteriori*,* but the philosopher in their armchair may talk to their colleagues, students, or pals at the pub, and the experimental philosopher may start with a set of assumptions, possibilities or cases generated from the comfort of their sofa. Nonetheless, we can (and do) make sense of the distinction, not least for the attendant weaknesses of each approach. For instance, philosophising from the armchair can lead to insoluble arguments: ‘Obviously this is what is meant by ‘*x’*’; ‘no, that’s not *really* what people mean by *x’*, and so forth. In addition, philosophers don’t tend to represent the average person on the street (Knobe 2007: p. 82): doing philosophy can skew one’s intuitions, there is a diverse range of views (among both the folk and philosophers) on many matters, and (Western analytic) philosophy has historically been (and still is) a male-dominated, overwhelmingly white profession (Goldman 2007; Machery et al. 2013; Nado 2014). Finally, there is growing evidence that philosophers are subject to biases and framing effects, albeit “in a slightly different manner than non-philosophers” (Nado 2014: p. 634). In short, in the armchair we’re limited by our biases, our education and, as I suggest below, our imagination.

X-Phi, by contrast, has the advantage of taking in more than just our own views or best guesses, employing “survey techniques to collect systematic data on the intuitions of large groups of subjects” (Nado 2014: p. 631). Nonetheless, there are well-documented priming and skewing effects depending on how questions are framed, studies are often expensive and time-consuming, and there are sampling issues: it’s difficult to get a good demographic distribution among participants (a lot of X-Phi thus involves undergraduate students). As Bernard Williams puts it, certain experiments lead the participant, eliciting a certain response because of how they are constructed:

It is the product of the will of the experimenter to produce a situation which would naturally elicit, with minimum hesitation, that description (1970: p. 79).

There have also been objections raised to the general survey approach (Ludwig 2007; Kauppinen 2007; Pust 2019).

The aforementioned challenges may be identified, suitably enough, from the armchair, but others only become obvious after the fact. For example, Nahmias et al. undertook a series of studies to capture free will-related folk intuitions, and encountered several unexpected problems. First, that “some participants seemed to fail to reason conditionally” (2005: p. 566): presented with a counterfactual scenario, participants wouldn’t entertain the antecedent to see if the consequent followed. When asked, ‘if there was a supercomputer who could predict the future, would Jeremy be free?’.

Some seemed to assume that the scenario is impossible *because* Jeremy has free will rather than making judgements about Jeremy’s freedom on the assumption that the scenario is actual (2005: p. 574).

(Many of us have encountered a similar problem when teaching first-year classes).

They also struggled with how to make concepts like ‘determinism’ accessible, without either using the technical term (as people tended to attach additional fatalistic assumptions to it) or needing to provide too much background information and instruction (for instance what ‘propositions’ or ‘entailment’ are). They write,

In our attempts to make determinism salient to participants, we described scenarios that many found implausible or impossible. Despite our efforts to induce participants to make their judgements based on a conditional acceptance of the scenario, it is likely that some did not do so. Hence, the challenge is to describe determinism in a way that participants find salient, intelligible, and somewhat believable (2005, p. 574).

A common feature of both the armchair method and X-Phi are their use of thought experiments to elicit intuitions. Liao and Gendler (2019) note that it is “incontrovertible that imagination is central to thought experiments.” But while they are referring to what goes on when we are presented with them (more on this in Sect. 4), there is another sense in which imagination is central to thought experiments: it is required for their creation. It is little wonder that a lone philosopher in the armchair might be limited by their imagination, but the same is true of X-Phi; even the most carefully constructed studies are limited by the scenarios we (usually philosophers, with our attendant weaknesses) dream up to pose to the test subjects. I propose a third way, a new intuition-capturing methodology not to replace, but to complement, the armchair and X-Phi, that overcomes some of the aforementioned weaknesses and utilises the imagination of the many rather than the few.

## The third way

Our current methods depend directly on people and what they report their intuitions to be: the philosopher in the armchair, or the test subjects in an X-Phi study. The methodology I propose instead looks at artefacts: patterns in what people have made.^4^ More specifically, it consists of identifying and appealing to ‘tropes’ in popular media.^5^ Tropes are recurring patterns, motifs or ideas, and manifest across any media where stories are told: films, television, literature, video games, web comics, and so on. Popular literary tropes—the context in which the term is most often used—include the familiar ‘damsel in distress’, ‘knight in shining armour’, and ‘love triangle’. I’m concerned with a less commonly discussed subset of tropes that pertain to areas of philosophical interest: for instance, ‘you can’t change the past’, ‘free will requires choice’, ‘the person goes with the mind, not the body’.^6^ (I’ve made use here of examples from metaphysics, but the approach applies across the board.)

These tropes need not tell us what people *believe* (any more than an intuition need tell us what people believe^7^—although they might); for the sake of the methodology something much weaker is required. Fiction, like thought experiments, presents us with imaginary scenarios—allowing us to “represent possibilities other than the actual, to represent times other than the present, and to represent perspectives other than one’s own” (Liao and Gendler 2019). I propose that tropes reveal which theories, concepts and ideas we find intuitive—repeatedly and *en masse*—in those scenarios, whether that be cashed out in terms of what we judge or what appears to us to be true, plausible or possible; what we are inclined to believe (or so on as your theory of intuitions dictates). If an idea is too unintuitive, it does not survive to become a trope.^8^

It may be the case that something about what is logically or conceptually possible could be derived from the stories we can imagine and/or the tropes that emerge, but that is not my claim here.^9^ Whether intuitions correspond with truth—no matter how they are gathered—is a question for subsequent philosophical analysis, not the intuition gathering stage. However, it would not be outrageous—and would be compatible with many accounts—to suggest that tropes provide insight at least into what people deem to lie within the boundaries of possibility.^10^ Indeed, as is familiar, several popular modal arguments rely on the stronger claim “that what one can imagine functions as a fallible and defeasible guide to what is really possible in the broadest sense” (Liao and Gendler 2019), and tropes are a subset of the imaginable: they indicate what people are repeatedly and popularly willing and able to imagine. Nonetheless, my proposal here is just that trope analysis identifies—and in some cases tropes correspond directly to—folk intuitions.^11^ (I discuss concerns about whether folk intuitions gleaned from fiction are useful or sufficiently reliable in Sects. 5.1 and 5.2).

Imagination plays a dual role in all three methods of intuition gathering: it allows us to create the thought experiments or fictions, and it allows us to mentally represent the latter, thereby eliciting the intuitions. My approach allows us to leverage the imagination of the many rather than the few on both counts: scenarios are both created and consumed by the folk themselves.

The best way to understand how the approach works is to look at examples, so I shall start with those, and then in Sect. 4.1 say a bit more about the mechanics. In Sect. 4.2, I highlight some advantages, and in Sect. 4.3 show what’s novel about the methodology. Finally, in Sect. 5, I consider objections.

Sometimes there is a predominant trope relating to a given question or concept that pervades texts across time, for instance, the idea that a foreknown future isn’t free (note that the first two examples below are not from fiction, but are illustrative of where philosophy and fiction have agreed—as we shall see, this is not always the case):

[I]f all things have been foreknown: and if they come to pass in this order… then by fate… all things happen which happen. But if this be so then there is nothing in our power and there is no such thing as freedom of will; and if we grant this, says [Cicero], the whole economy of human life is subverted (Augustine 2006: Bk V Ch. 9 §2).

I don’t see how God can have foreknowledge of everything and that there can still be free will. If God sees everything that will happen, and if he cannot be mistaken, then what he foresees must necessarily happen. And if he knows from the very beginning what all eternity will bring, not only men’s actions but their thoughts and desires will be known to him, and that means that there cannot be any free will (Boethius 2008: Bk V Ch. III).

Oracle: Candy?

Neo: Do you already know if I’m going to take it?

Oracle: Wouldn’t be much of an Oracle if I didn’t.

Neo: But if you already know, how can I make a choice? (Wachowski and Wachowski 2001).^12^

Recall the problem that Nahmias et al. discovered (Sect. 3): getting people to entertain the existence of a foreknower. Fiction overcomes this hurdle; one expects that the audience watching *The Matrix: Reloaded* is willing to accept that (a) there could be such a thing as the oracle (it is conceptually coherent), and that (b) that thing could be a computer program (as the plot describes). The extent of the audience’s willingness is evidenced by the recurrence of both oracle or prophet type characters in fiction, and the pervasive appearance of sentient software: i.e. they’re sufficiently tropey.^13^ The more interesting trope for my purposes is that exhibited by Neo’s response: that if someone knew in advance what you would do, then you wouldn’t be free to choose. This intuition is at odds with many contemporary philosophers who work on the subject, but it continues to pervade the popular canon.

That’s not to say that this is the only trope relating foreknowledge and free will. The point isn’t to find the one definitive folk intuition: people are diverse, as are their views.^14^ But insofar as there are ‘common-sense’ views on the matter, this one—that a foreknown future is at odds with our free will—is widespread.

Sometimes the tropes reveal a cluster of different views, for instance, different conceptions of time in time travel texts. Most fall neatly into one of two categories: those that posit a dynamic timeline (in which you can change the past) and those that posit a static timeline (in which you cannot).^15^ Thus both ‘time travellers could change the past’ and ‘time travellers could not change the past’ are tropes (this should be neither surprising nor worrying: philosophical theories are often similarly at odds).

In the time travel case, the tropes are a feature of the worlds of the respective fictions, but tropes needn’t be in-built like this; instead they might be expressed as views of the characters. So, for instance, we find opposing answers to the persistence question with regards to the importance of memory in preserving personal identity:

“If you take away what they know, you take away who they are.”

– Four, *Allegiant.*

“This is Caroline. Minus the memories, but it’s her and this is exactly what Caroline would do.”

– Adelle Dewitt, *Dollhouse* S01E08.

Whether these opposing responses are tropes depends on their recurrence and pervasiveness (so, for instance, ‘vampires sparkle’ is not a trope—despite the popularity of the specific series in which they do—but ‘vampires can be killed with a stake to the heart’ is).^16^

### The mechanics

Nadelhoffer writes, “the only method of determining what the majority of nonspecialists say about particular cases is to actually ask them” (2004: p. 202). Trope analysis suggests otherwise. In rough and ready terms, the approach is this: instead of just thinking about what is meant by a concept or idea, or asking people what they mean by it or what they think is meant by it, look for what people have said/written/made/engaged with about it. Philosophers already do something like trope analysis when summarising the state of play on a given philosophical topic, making claims like ‘there are two broad camps in the literature’, or ‘philosophers have tended to claim that *p’;* I’m suggesting we broaden the net. In one sense the proposal is very simple: rather than asking people—as Nadelhoffer suggests—one looks at what they’ve said unbidden, at what they’ve created and consumed.

The difference between typical X-Phi and this new approach is akin to the difference between a clinical trial and an observational study in medicine: in trope analysis, as in the latter, the focus is on what happens in ‘the wild’, amongst the folk.^17^ The environment and data are not so easily controlled—there are no stipulated thought experiments with “crucial characteristics…highlighted for the subject, to focus attention on what is relevant to the general account currently being tested” (Goldman 2007: p. 15)—but a wider net is cast. There are some purposes that favour armchair over experimental philosophy and vice versa; so too trope analysis lends itself to some purposes more than others. It is a particularly useful tool when mapping the landscape of possibility around a given concept, checking one’s usage against the folk’s, or determining if there’s a disconnect between the philosophical canon and the spectrum of folk intuitions. Confirmation that a particular case satisfies a candidate theory, by contrast, may be more efficiently gained through one of the existing methods (unless there happens to be a fictional equivalent of the case).^18^ Given the range of purposes to which folk intuitions are put, it is unsurprising that three tools might be better than two.

The biggest challenge for those utilising this methodology is the identification of relevant tropes, but they need not start from scratch; there is a great deal of data already collated. The first and most immediately useful source is the online wiki ‘TV Tropes’, where tropes and their instances are catalogued. Therein we find tropes corresponding to those discussed above: you can’t change the past; you can change the past; a foreknown future isn’t free, and so on. The second are corpora such as the British National Corpus, the German National Corpus or the Corpus of Contemporary American English. Assembled by linguists, these are most helpful for those ideas that lend themselves to a keyword search (e.g. free will; time travel). Corpus analysis is not tantamount to trope analysis, but can be a useful starting point for finding texts relevant to a given enquiry.^19^ As with all data, that gathered from the aforementioned sources needs to be carefully scrutinised to ensure its accuracy and pertinence; sometimes ideas, usages or accounts that would be distinct to a philosopher are conflated. Nonetheless, these starting points can cut down the time and media consumption required to get an overview of the tropes in a given domain.

As noted earlier, but worth re-emphasising, the identification of tropes isn’t the end of the story; it’s the beginning, after which we commence our traditional philosophical analysis. It is a method for getting more data about folk intuitions, checking that our spectrum of theories contains as many live options as possible, and ensuring that we’re doing philosophy that is relevant to the matter with which we take ourselves to be concerned.

### Advantages

Trope analysis is a useful tool in the folk-intuition-seeker’s toolkit. Although best viewed as a complement to existing approaches, it does have some advantages which recommend its use. Given a sufficient range of texts and tropes, the data is more abundant, leading to a more comprehensive map of conceptual space, and less vulnerable to some priming and framing effects (see also Sect. 5.3).^20^ Text creators being more diverse than philosophers, and text consumers even more so, tropes are representative of a broader range of views than the traditional methods (experimental philosophers motivated by concerns about the representativeness of philosophers’ intuitions and their relative homogeneity versus the diversity of the folks’ should thus be sympathetic to my approach).^21^ Yet trope analysis doesn’t require a travel budget or ethics approval, and can largely be conducted from the comfort of the armchair. Trope analysis additionally allows for diachronic study of intuitions dating back before the commencement of a given investigation.^22^

One of the objections levied against X-Phi surveys is that control questions—used to ensure conceptual competence on the part of the subject—“amount to presupposing that certain answers will not reflect the folk concept” (Kauppinen 2007: p. 106). Experimentalists whose arguments depend on variation in intuitions (e.g. Machery et al 2013) might find trope analysis beneficial as an initial step in determining the general landscape of folk intuitions so as to ensure their control questions do not inadvertently rule out particular intuitions live among the folk.

Using fiction also overcomes the two challenges highlighted by Nahmias and colleagues discussed in Sect. 3: getting participants to think counterfactually (as discussed above) and making technical concepts more accessible (as evidenced by the tendency of philosophers to use fictional examples to illustrate ideas). At minimum, then, trope analysis could be combined with existing methods to help us build more plausible thought experiments, allowing participants to entertain antecedents more readily, and—thanks to the greater number of creators working on fictional scenarios—giving us the best chance of making ideas salient without the use of technical terms. As Ichickawa and Jarvis write,

We can use fictional texts to communicate far more than their literal contents. This in turn allows people to come to grasp propositions too difficult to easily express in literal speech (2009: p. 235).

Both of our current methods are restricted by our imaginations: what thought experiments we can dream up to elicit intuitions, either from ourselves or from study participants. Trope analysis allows us to leverage the imaginations of many, indeed of the folk *en masse.* It also enables philosophers to identify and engage with ideas that have captured the popular imagination, which for many of us is an additional boon.^23^

### Used but new

Philosophers attending to fiction is not an unusual occurrence, but broad-scale, cross-media trope analysis is novel in philosophy, despite haven proven fruitful in other disciplines (dating back to Aristotle’s *Poetics)*. Philosophers have analysed individual texts (e.g. *The Blackwell Philosophy and Pop Culture Series)* or used examples from fiction to illustrate a theory (e.g. Hanley 2004). They have engaged in philosophical analyses of fiction itself (e.g. truth in fiction—Lewis 1978) and the implications of our emotional or moral responses to it or its characters (e.g. Camp 2009: p. 107). Speaking broadly, rigorous philosophical engagement with fictional texts has tended to be theory-led, asking what texts reflect or illustrate a given theory, how we might understand a text through the lens of a given theory, or more abstract questions about the status or mechanics of fiction. These are legitimate questions to explore, but they don’t exhaust the usefulness of fiction to our philosophical endeavours. Thus a second novelty of the proposed approach is that it is, as much as possible, text-first. This permits room for ideas and theories lying outside the extant philosophical canon, but still within the spectrum of possibility; it also allows the recognition of subtleties in texts (and across texts, given trope analysis) that only become evident when we stop looking through the lens of our preferred theory.^24^

Finally, and most significantly, it is novel to use trope analysis for the purpose I propose: to identify folk intuitions.

But with novelty comes doubt. In the following section I respond to potential objections to the use and usefulness of the method. I have grouped the bulk of these into two clusters: (1) worries about deriving folk intuition data from fiction (Sect. 5.1) and (2) worries about deriving intuitions from speculative fiction in particular (Sect. 5.2). Then, I consider a concern about the direction of influence between tropes and intuitions (Sect. 5.3).

## Objections

### Impossible fictions and conceptual falsehoods

#### Objection: Can’t there be impossible fictions?

This objection dates back at least to Descartes, who observed that “fiction makes us imagine a number of events as possible which are really impossible” (1950: p. 5). One doesn’t have to look far to discover impossible fictions—time travel stories, for instance, are frequently plagued by logical inconsistencies.^25^ However, not all parts of an impossible fiction are impossible, and as far as the method is concerned only the tropes are of interest. Thus most impossibilities won’t enter into our dataset, because the impossible circumstances won’t be replicated sufficiently to become a trope. However, if the tropes themselves are impossible—as we might think ‘you can change the past’ might be, depending on our background theory of time—then that’s really interesting! (And would motivate us to investigate what it is about the trope that makes it so pervasive despite its impossibility, as we might for mistaken intuitions identified in other ways).

Of course this same worry can be levelled against thought experiments. As Ichikawa and Jarvis ask, “How can we know that the story we’re engaging with in a thought experiment describes a possible situation?” (2009: p. 233). In either case, the burden is then on the philosopher to identify the impossibility and to articulate (and perhaps suggest an explanation for) the disconnect between reality and common sense.

#### Objection: conceptual falsehoods

However, even if we’re not worried about *logically* impossible stories, we might have a related and arguably more serious concern: what about *conceptually* impossible stories? Although I have tried to remain neutral with respect to what intuitions serve as evidence or justification for, we might think that for the intuitions (and the method for accessing them) to be useful, they should—at minimum—reveal how people use concepts and what they take the boundaries of possibility to be. Given this, one might worry about people entertaining conceptual impossibilities.

Van Inwagen proposes an objection of this sort in a paper on mereology, where he notes that he’s not going to tackle one of his interlocutor’s points, because

Examples drawn from literary fantasy are essential to his argument, and, in my view, one may not use examples from fantasy in conceptual investigations. The reason is simple: the author of a fantasy has the power to confer “truth in the story” on known conceptual falsehoods. I could, for example, write a fantasy in which there were two mountains that touched at their bases but did not surround a valley. *A fortiori,* the author of a fantasy has the power to confer truth in the story on a proposition such that it is a controversial philosophical question whether that proposition is a conceptual falsehood (1993: pp. 229–230).

It’s worth separating out two different concerns here:

1. Does the possibility of conceptually impossible stories—or those containing conceptual falsehoods—affect the usefulness of the method for gathering folk intuitions?
2. Does this possibility affect the usefulness of the intuitions for subsequent conceptual analysis?

The answer to (1) is straightforward. The goal of the methodology is not to work out what truths the story contains^26^ or whether concepts are being used appropriately—that is part of the next step, the subsequent philosophical analysis, rather than the intuition gathering. At this initial stage, we’re concerned with what is contagious, compelling and pervasive, rather than what’s true or reflective of reality. (You might worry that stories with conceptual falsehoods have an undue influence on folk intuitions—I deal with this in Sect. 5.3).

As for (2), it may be the case that some fiction plays with concepts in a way we would deem ‘misuse’—a classic (if somewhat outdated) example might be ‘some mothers are male’.^27^ We can say many of the same things about such cases as I offered for impossible fictions: if the examples are isolated anomalies, they won’t make it into the dataset (as they won’t become tropes); however, if a ‘conceptual falsehood’ were to survive to become a trope, then perhaps we should reconsider whether it is genuinely a conceptual falsehood (as opposed to a conceptual shift, the concept being broader than we had realised, or a new concept using the same word). Even if there are cases of mass confusion, that in itself is interesting: what makes them so tenacious?

But we can go slightly further. Although I am not committed to the claim that folk intuitions are a guide to the actual way things are, or the way they might (metaphysically or logically) have been—as opposed to what people take to be possible—I am sympathetic to the following from Ichikawa and Jarvis:

There does seem to be a useful notion of conceptual possibility to which this conceivability is an excellent guide. Conceptual possibility is closely tied to what one can rationally and coherently conceive. If a proposition is a conceptual possibility, then an ideal rational agent can coherently conceive of it as true… (2009: p. 233).

Like them, I find compelling the notion that there’s a kind of possibility—perhaps an imaginative possibility—that picks out something interesting and useful. Importantly, this possibility has limits: some ideas an audience refuses to entertain, and others last only the length of a film or series (e.g. sparkly vampires). Tropes reveal those ideas that multiple people repeatedly find intuitive; they are artefacts of our popular imagination.

But even if the objection goes through and some stories (and even some tropes) contain conceptual falsehoods, clearly not all (or even most) are problematic in this way. Fictions “are (at least typically) generated so as to maintain their collective coherency” (Ichikawa and Jarvis 2009: p. 234)—they are very hard to imagine otherwise. Some fictions not only pass the imaginability test but are deemed internally consistent by philosophers, as Lewis notes in “The Paradoxes of Time Travel”:

Not all science fiction writers are clear-headed, to be sure, and inconsistent time travel stories have often been written. But some writers have thought the problems through with great care, and their stories are perfectly consistent (1976: p. 145).

### Speculative fiction and intuitions about distant worlds

#### Objection: even if tropes give insight into folk intuitions, why should we trust our intuitions about stories that take place in worlds very different to our own?

Something like this objection has been levelled against certain thought experiments, so if it can be overcome, that’s a boon not only for my approach but for the armchair and X-Phi as well. Nahmias et al.’s free will X-Phi has been criticised for “asking the folk to consider highly extraordinary stories” about circumstances very different to what they have experienced:

[I]t is very difficult to intuitively prescribe whether [an agent acts freely] when the context is out of the ordinary; or when our beliefs are being questioned… One cannot appeal to common sense while challenging it (Gasparatou, 2010: p. 380; Cf. van Inwagen 1998: p. 70).

Quine objects similarly to the use of ‘absurd’ science-fiction scenarios when contemplating personal identity:

To seek what is “logically required” for sameness of person under unprecedented circumstances is to suggest that words have some logical force beyond what our past needs have invested them with (Quine 1972: p. 490; Cf. Gendler 2002).

If compelled by this, and borrowing the familiar notion that thinking about fiction or thought experiments is akin to counterfactual thinking (Lewis 1978; Camp 2009; Williamson 2008),^28^ one might think that our thought experiments and the tropes we investigate should be limited to the closest possible worlds (or fictions that take place in worlds like our own). However, it’s not clear (a) that we can’t learn from distant possible worlds, or (b) that the worlds of speculative fiction are that distant or the scenarios they depict so ‘extraordinary’. To quote Parfit’s (1984) response to Quine,

This criticism might be justified if, when considering such imagined cases, we had no reactions. But these cases arouse in most of us strong beliefs. And these are beliefs, not about our words, but about ourselves.

Ichikawa and Jarvis ask us to entertain a case where a planet’s oceans are filled with orange juice and someone suffocates when their lungs fill with juice upon being submerged. They ask,

Did that person drown? Most English speakers will answer that he did. In this way we might find evidence that the particular liquid a person suffocates in is immaterial to whether he or she drowns—it would seem we have succeeded in doing some conceptual analysis of drowning (2009: p. 238).

A world with orange juice oceans is arguably more distant than many of the worlds of speculative fiction, yet Ichikawa and Jarvis make claims significantly stronger than mine on the basis of our intuitions about it.

The most creative, original fiction still holds fixed “part of our factual background”—it is from there that we can “safely reason” (Lewis 1978: p. 170). Speculative fiction has spawned a plenitude of pervasive tropes, some of which have become decidedly ordinary. Worlds, and stories, may be far away without being far-fetched.

#### Objection: what we find intuitive in the context of speculative fiction is different to what we find intuitive in our everyday lives

It seems true that my intuitions might vary from case-to-case depending on context. Suppose that I see a blue police box and hear a ‘vworp’ noise. The explanation I would find most intuitive if I was watching television at the time (that it was a time traveller arriving on screen) is quite different than if I was standing in the main street of town (where the police boxes are used as convenience stands and any strange noises are to be attributed to buskers).

If this is a problem—and it’s not clear that it is—it is not unique to trope analysis. Gendler (2007), for instance, argues that our abstract intuitions often differ from those elicited via thought experiments. There are clear examples of this: the tripartite (JTB) theory of knowledge is intuitive to many, and yet so too are the Gettier cases which indicate the theory’s insufficiency. Earlenbaugh and Molyneux present a convincing account of this and why it’s unproblematic in terms of competitive intuitions: we have “one collection…that concern cases and another…that arise from general principles or theory” (2009: p. 107 fn. 20). Likewise, we may have one collection of intuitions at play when contemplating what the world would be like if there were time travellers (such as one might when writing an episode of *Doctor Who*)—which might in turn affect other intuitions, like those pertaining to our concept of time—and another when we think about time in the abstract or time in our everyday lives (which we don’t usually think involves time travel).

Returning to the Gettier cases, Nagel notes that “as counter-examples became more elaborate—involving subjects with strange new perceptual faculties or paranormal powers—it was also found that these cases did not always trigger the sort of robust and widely shared responses that greeted Gettier’s original examples” (2007: p. 797). One advantage of trope analysis over single thought experiments is that tropes *are* ‘widely shared responses’, even when they pertain to ‘subjects with strange new perceptual faculties or paranormal powers’, time travel, or prescient programs.

### Worries about direction

#### Objection: folk intuitions should come from the folk—why think that this method reveals the intuitions of the folk rather than just the intuitions of the media creators?

It is plausible that our intuitions are shaped by our experiences and circumstances (including, but not limited to, the media we consume); they don’t occur in a vacuum.^29^ Media doesn’t exist in a vacuum either: the experiences and circumstances of its creators influence it, but so do concerns about what the audience will accept, enjoy and engage with. I’m happy to concede that engaging with particular types of media might influence one’s intuitions and expectations, but suggest that the causal influence goes both ways, with media responding to uptake. As part of this process, some ideas repeatedly emerge and thus become tropes.^30^

Of course, if only a few voices dominate text production, then we might worry that trope analysis would be a less effective measure of the intuitions of the masses, since the texts would be limited by the imagination of a few. But this is no longer the case: thanks to the internet and alternative methods of publishing, a much larger range of creators are enfranchised to both produce and distribute their work.^31^ Some of the most interesting trope subversions and developments have arisen from the amplification of minority voices, through self-publishing, independent games, web comics and so forth. Looking at tropes in a range of texts helps capture a diverse range of intuitions that better reflect the diversity of the folk.

## Conclusion

It is striking that so many of the objections to using tropes in fiction to derive folk intuitions—potential impossibility, conceptual falsehoods, extraordinariness—can also be levelled at the thought experiments that underpin much of both armchair and experimental philosophy. Formulating responses to them is thus a positive step for the philosopher’s toolkit as a whole, not just the new methodology I propose. However, there are some advantages to the latter that provide additional motivation for its use, not least because fiction is full of rich, complex, diverse thought-experiment-like scenarios that philosophers didn’t write, and thus that don’t have our limitations. A weaker conclusion, then, might be that we should make more use of fiction in our experimental philosophy and armchair musings, in addition to (or in some cases instead of) the thought experiments we devise.

But this isn’t the only reason we should add trope analysis to our philosophical toolkit. It also has the benefit of providing a new way for academic philosophy to access and interact with questions of perennial folk interest, beyond treatments of individual texts or theories. In helping us to identify a greater range of folk intuitions, trope analysis furthers our philosophical understanding, providing new data for conceptual mapping and analysis. And finally, the approach reveals the promise of philosophy to help us tell new stories, shining light on and making clear existing patterns and thereby encouraging the transcendence and subversion of current tropes. Eliciting folk intuitions via cases has always involved harnessing the imagination, both in their construction and in their use; trope analysis allows us to widen our net to capture the imaginative outputs of the many, rather than the few.

## Footnotes

1 For further examples see Kripke (1980: p. 42), Goldman (2007: p. 1) and Williamson (2004: p. 150).

2 There are several debates on this front, including whether the intuitions of the folk or of philosophers serve as better evidence (e.g. Goldman 2007; Kornblith 2007), what intuitions serve as evidence of or justification for (e.g. Bealer 1998; Kornblith 2007; Williamson 2004), whether intuitions *should* be used as evidence (e.g. Earlenbaugh and Molyneux 2009), and whether intuitions *are* actually used as evidence (e.g. Deutsch 2015; Cappelen 2012). For an overview, see Pust (2019) and Nagel (2007).

3 Clearly this does not exhaust the potential of either the armchair or experimental philosophy, but in this paper when I speak of the armchair or X-Phi I’m referring to accessing folk intuitions specifically.

4 There is an analogy to be drawn here with linguistics, which makes use of linguists’ own intuitions (analogous to the armchair), surveys (X-Phi), and corpus analysis (looking at artefacts). See also fn 19.

5 For the examples in this paper, I’ve concentrated on speculative fiction (fantasy and science fiction), for two reasons: (1) as a genre it lends itself to the exploration of the kinds of concepts at stake in the literature cited (free will; time; knowledge etc.), and (2) there are more objections levied against the use of fantastic stories/examples at distant worlds than against other types (see Sect. 5.2)— if these can be overcome, then the approach as a whole is on firmer footing. Other genres might prove more useful for other areas of philosophy; the approach is transferable.

6 Of course, even the aforementioned literary tropes are philosophical, insofar as they tell us about gender and sexuality norms (and how they change over time).

7 For instance, Earlenbaugh & Molyneux (2009) argue that intuitions are neither belief- nor credence-entailing.

8 Where ‘too unintuitive’ means intuitive to too few people, or in too few contexts/circumstances. Tropes are not *by definition* those ideas that people find intuitive, but rather there is a correlation between the two: tropes reflect the intuitions of the folk, and ideas that are ‘too unintuitive’ thus lack corresponding tropes.

9 It is evident that at least in some cases there is a difference between where the folk draw the boundaries of possibility and where philosophers would argue they in fact lie. See Sect. 5.1 for more on the discrepancy between conceptual possibility and folk intuitions/tropes.

10 For this and stronger positions, see for example Chudnoff (2011: p. 625), Nagel (2007: pp. 792, 804), Kornblith (2007) and Williamson (2016).

11 If it is a (philosophical) trope that *p,* then it is a folk intuition that *p.* However, many tropes are not formulated as propositions, at least without further analysis. This is not a concern; the same appears to be true of folk concepts, which empirical work suggests “are not usually represented in us as sets of necessary and sufficient conditions, but rather by means of prototypical examples together with a multi-dimensional range of ways and degrees to which members of the category can deviate from the prototype” (Nagel 2007: p. 804). Where tropes present as propositions they correspond neatly to folk intuitions; where they do not, further analysis allows us to identify the intuition underpinning the trope. The ‘philosophical’ delineation is not precise, but there are some tropes—particularly those relating to genre- or medium-specific mechanics rather than narratives (e.g. those pertaining to types of video game level)—that reveal limitations or conventions of the medium rather than folk intuitions; I am not including these under ‘philosophical’.

12 Other examples include “My life isn’t my own. I’m a puppet for the Pattern and the prophecies, made to dance for the world before having my strings cut… my choices are made for me by the Pattern itself” (Robert Jordan and Brandon Sanderson, *The Gathering Storm* Kindle Edition, (Orbit, 2010) Ch. 47); “If I am granted a vision of the future, I will not try to prevent anything that I see. It never works” (John Van Sickle “Things I Will Do If I Am Ever the Hero” (2005), http://www.geocities.ws/evilsnack/hero.html).

13 You might worry that audiences are willing to entertain even the impossible—I address this in §4.

14 As X-Phi has demonstrated e.g. Weinberg et al. (2001) and Machery et al. (2004).

15 In terms of films, *Twelve Monkeys* and *Bill and Ted’s Excellent Adventure* fall in the latter category, and *Looper* and *Back to the Future II* the former. Video games also exhibit this dichotomy—*Day of the Tentacle* and *Dragon Age Inquisition* present a dynamic timeline, and *Jak and Daxter* a static one—and so does television (e.g. *Doctor Who* is frequently dynamic, but certain episodes, e.g. ‘Blink’, are static) etc.

16 Vampires sparkle in the *Twilight* series*.* Death-by-stake occurs in numerous texts across different media, including *Dracula; Shiki; The Vampire Diaries; True Blood; Buffy the Vampire Slayer; Castlevania: Lords of Shadow* and *Baldur’s Gate II.*

17 It has been remarked of clinical trials (in comparison with observational trials) that they focus “on an assessment of the validity of [their] results at the expense of generalizability” (Bokyo 2013)—the same might be said for X-Phi versus trope analysis.

18 Kauppinen observes that two ways X-Phi surveys are used are “to settle whether people really have the sort of intuitions that philosophers assume they do” and “to challenge conceptual assumptions that philosophers routinely make” (2007: p. 99). X-Phi is excellently positioned to do the latter (see for instance Knobe’s (2003) comparison of reactions to two cases differing only by an ostensibly irrelevant variable); even should the tropes align to permit trope analysis to attempt the same, we should not expect this to be possible in every case (or even most cases), and it’s unlikely to be the most efficient method. However, trope analysis provides a cheap, effective way of doing the former, i.e. settling whether people have the intuitions philosophers think they do; it is a particularly useful way of working out the intuitional landscape.

19 The use of corpus analysis is growing in philosophy; as a source of “empirical linguistic data” corpora are particularly useful for “answering language-related research questions” (Bluhm 2016). Trope analysis—where the tropes in question are philosophically substantive (as opposed to linguistic tropes)—is not reducible to corpus analysis, as tropes are not always indicated by a certain keyword or set of phrases. As Goldman (2007: p. 18) warns, “it would be a mistake to equate the domain of *conceptual* analysis with the domain of *linguistic* analysis”.

20 In addition to those mentioned in Sect. 2, another potential confounding factor in X-Phi is difference in linguistic background (Nagel 2007: p. 801), i.e. how test subjects interpret certain words in the questions they’re asked. Looking at a diversity of texts from creators of different linguistic backgrounds—not just in terms of different languages or dialects, but also that use different registers—might help us identify or minimise such instances.

21 To capture the full diversity of text creators and consumers, it’s important to include not just mainstream films and novels from prestige presses in our trope analysis—see Sect. 5.3.

22 For instance, here in 2020 I can get a picture of how folk intuitions about time and time travel have changed since the publication of H. G. Wells’s *The Time Machine,* without needing my own time machine.

23 This is another respect in which experimentalists should be sympathetic to trope analysis. For instance, Knobe writes that if he had to explain his research, he’d say “I study the most basic concepts people use to understand themselves and their world” and notes that his fellow X-Phi proponents “don’t understand how certain philosophers can fail to be moved by issues that other people find so deeply important” (2007: p. 90). Trope analysis helps us identify ideas, theories and questions that repeatedly capture the folk imagination, and does so by examining the artefacts in which people have explored themselves and their world.

24 These concerns are also relevant to the construction of thought experiments—often we start with a set of possible answers. Those may not reflect the full spectrum of possible answers, and our construction may be skewed, as suggested above, by our pre-existing theoretical commitments.

25 Any of the ‘dynamic timeline’ stories mentioned in fn. 15 arguably fall into this category.

26 Lest it be ambiguous, I mean here ‘what (objective) truths there are in the text’ as opposed to ‘what is true in (the context of) the story’.

27 We see the inverse of this in the *Mass Effect* series—the asari species can procreate with any gender or species, but refer to ‘fathers’ as the parent who didn’t give birth to the child. So, in the text, ‘some fathers are female’ is true.

28 Liao and Gendler (2019) also note that imagination—which allows us to represent the scenarios in fiction and thought experiments—is also what “lets one move from counterfactuals’ antecedents to their consequents.”.

29 Evidence suggests that intuitions are affected by a number of factors, including (but not limited to) cultural background, socio-economic status, gender, order of presentation, personality traits, education, theory adoption, whether there is a clean smell in the room, and so on. For an overview see Nado (2014).

30 An anonymous reviewer notes that there may be cases where ideas are frequently perpetuated and then acceptance follows. One example might be, I suppose, stereotypes in ‘fake news’/propaganda. That might very well be true, and points to the importance of looking at a diverse range of texts over different media and by different creators. Additionally, as noted above, I think it’s particularly the case in fiction that the influence between texts and consumers (and thus the tropes in the former and the intuitions of the latter) goes both ways (thanks to the internet as a forum for dialogue between creators and consumers, alternative methods of publishing allowing for greater overlap between the two etc.); the correlation between the two allows us to derive information about intuitions from tropes.

31 Fanfiction is a notable example and worth including in trope analysis: fanfics as a medium have their own tropes, as well as sharing some with the canonical texts that inspire individual works.
